# Probabilistic-Input, Noisy Conjunctive Models for Cognitive Diagnosis

**DOI:** 10.3389/fpsyg.2018.00997

**Published:** 2018-06-14

**Authors:** Peida Zhan, Wen-Chung Wang, Hong Jiao, Yufang Bian

**Affiliations:** ^1^Collaborative Innovation Center of Assessment Toward Basic Education Quality, Beijing Normal University, Beijing, China; ^2^Assessment Research Centre, The Education University of Hong Kong, Tai Po, Hong Kong; ^3^Measurement, Statistics and Evaluation, Department of Human Development and Quantitative Methodology, University of Maryland, College Park, MD, United States

**Keywords:** cognitive diagnosis, probabilistic logic, PINC model, DINA model, higher-order model, cognitive diagnosis models

## Abstract

Existing cognitive diagnosis models conceptualize attribute mastery status discretely as either mastery or non-mastery. This study proposes a different conceptualization of attribute mastery as a probabilistic concept, i.e., the probability of mastering a specific attribute for a person, and developing a probabilistic-input, noisy conjunctive (PINC) model, in which the probability of mastering an attribute for a person is a parameter to be estimated from data. And a higher-order version of the PINC model is used to consider the associations among attributes. The results of simulation studies revealed a good parameter recovery for the new models using the Bayesian method. The Examination for the Certificate of Proficiency in English (ECPE) data set was analyzed to illustrate the implications and applications of the proposed models. The results indicated that PINC models had better model-data fit, smaller item parameter estimates, and more refined estimates of attribute mastery.

## Introduction

Unlike item response theory (IRT) models, which locate an examinee's latent trait on a continuum, the purpose of cognitive diagnosis models (CDMs) is to classify an examinee's latent attributes into a set of binary categories. The output of the analysis with conventional CDMs is a profile with binary outcomes (either 1 or 0) indicating a person's mastery or non-mastery of each attribute. The binary classification follows standard or ordinary logic in that every statement or proposition is either true or false without uncertainty, which is referred to in this paper as deterministic logic. However, things are rarely black and white. A fundamental aspect of the human condition is that no one can ever determine without uncertainty whether a proposition about the world is true or false (Jøsang, 2001).

In contrast to deterministic logic, the aim of probabilistic logic is to integrate probability theory to handle uncertainty with deductive logic, in order to exploit the structure of formal argument (Nilsson, 1986; Jøsang, 2001). Probabilistic logic is a natural extension of deterministic logic, indicating that the results it defines are derived through probabilistic expressions. Specifically, a statement *S* (e.g., person *n* masters attribute *k*) is either true or false. There are two sets of possible worlds, one set (*W*_1_) containing worlds in which *S* is true, and the other set (*W*_2_) containing worlds in which *S* is false. Let the probability that our actual world is in *W*_1_ and *W*_2_ be *P*_1_ and *P*_2_, respectively, and *P*_1_ + *P*_2_ = 1. Because the truth-value of *S* in our actual world is unknown, it is convenient to imagine that the truth-value of *S* is the probability that our actual world is in *W*_1_, which is *P*_1_ (Nilsson, 1986). In this example, the statement “person *n* masters attribute *k*” is probabilistic rather than deterministic, and the probability is *P*_1_. Probabilistic logic has been widely used in computer science, artificial intelligence, and machine learning (Dietterich et al., 2008; Haenni et al., 2010). Also in the area of psychological and educational measurement, the IRT models that using logistic (or normal ogive) function to describe the probability of a deterministic result (e.g., a correct or incorrect item response) are good examples of the probabilistic logic. Similarly, attribute mastery can be constructed in probabilistic logic rather than deterministic logic.

Probabilistic logic treats attribute mastery status with uncertainty. The resulting attribute profile report for each person, from the probabilistic logic perspective, is a vector of numbers ranging from 0 to 1 that specify the probability of mastering each attribute. Although both deterministic logic and probabilistic logic assume binary attributes, they differ in their assumptions about attribute status. The status can be known with absolute certainty in deterministic logic, while it is known with uncertainty in probabilistic logic. Apparently, probabilistic logic is less restrictive than deterministic logic and can provide a finer description of mastery status.

Among the existing CDMs, the deterministic-input, noisy “and” gate (DINA) model (Macready and Dayton, 1977; Junker and Sijtsma, 2001) is one of the most popular models. This study aimed to develop a general DINA model, called the probabilistic-input, noisy conjunctive (PINC) model, in which the probability of mastering an attribute for a person is a parameter, so the individual differences in attribute status can be quantified more precisely than when the mastery status is either 1 or 0 in the DINA model or other existing CDMs. Furthermore, the higher-order PINC (HO-PINC) model has been developed to account for the associations among attributes. The rest of the paper starts with a review of the conjunctive condensation rule (Maris, 1995) and the DINA model, followed by an introduction to the PINC and HO-PINC models and parameter estimation with the Bayesian approach. The parameter recovery of the new models was assessed with simulations. An empirical example is given to illustrate the applications and advantages of the new models.

## Conjunctive condensation rule and the DINA model

Let *Y*_*ni*_ be the observed response of person *n* (*n* = 1, …, *N*) to item *i* (*i* = 1, …, *I*), *x*_*nk*_ be the latent variable for person *n* on dimension *k* (*k* = 1, …, *K*), and α_*nk*_ be the binary variable for person *n* on attribute *k*, where α_*nk*_ = 1 if person *n* masters attribute *k*, and α_*nk*_ = 0 otherwise. It is because α_*nk*_ is either 1 or 0 that deterministic logic applies. The variable α_*n*_ is the vector of attribute mastery status for person *n*. The Q-matrix (Tatsuoka, 1985) is an *I* × *K* matrix with element *q*_*ik*_ indicating whether attribute *k* is required to answer item *i* correctly; *q*_*ik*_ = 1 if attribute *k* is required, and it equals 0 otherwise. The Q-matrix is a confirmatory cognitive design matrix that identifies the required attributes for each item.

A condensation rule specifies the relationship between latent variables and latent (ideal) responses (Maris, 1995). Among the various condensation rules, the conjunctive one is the most commonly used (Rupp et al., 2010). In principle, not every latent variable has to be defined for a particular latent response, so a confirmatory matrix (i.e., the Q-matrix) is needed to specify the relationships between the items and latent variables measured by each item. Using *C* as a generic symbol for a condensation rule, the conjunctive condensation rule can be expressed as follows:

$$\begin{array}{l} \eta_{ni}=C\left({\text{x}}_{n},{\text{q}}_{i}\right)=\prod_{k\;=\;1}^{K}x_{nk}^{q_{ik}} \end{array}$$

which means that the latent response η_*ni*_ is correct only if all the latent variables are 1 (i.e., *x*_*nk*_ = 1 for every *k*).

In practice, latent responses can be considered as necessary antecedent terms to the observed responses (Whitley, 1980; Maris, 1995). If the process is non-stochastic, the latent responses are identical to the observed responses. Since human behaviors are seldom deterministic (e.g., students may make careless mistakes or guess wisely on a test, which brings noise to the observed item responses), latent responses can seldom be transferred to observed responses directly (Tatsuoka, 1985). In psychometric models, a commonly used item response function of the relationship between the latent and observed responses can be expressed as follows:

$$\begin{array}{r} p_{ni1}=P\left(Y_{ni}=1|\omega_{ni},\Omega_{i}\right)=g_{i}+\left(1-s_{i}-g_{i}\right)\omega_{ni}, \end{array}$$

where *p*_*ni*__1_ is the probability of a correct response for person *n* to item *i*; ω_*ni*_ is the latent response of person *n* to item *i*; **Ω**_*i*_ = (*g*_*i*_, *s*_*i*_)′ is a vector of the parameters of item *i*, and *s*_*i*_ and *g*_*i*_ describe, respectively, the slip and guessing probabilities in a simple signal detection model for detecting a latent response ω_*ni*_ from noisy observations *Y*_*ni*_. In practice, a monotonicity restriction (*g*_*i*_ < 1 – *s*_*i*_) can be imposed (Junker and Sijtsma, 2001; Culpepper, 2015). Note that the η_*ni*_ in Equation 1 is just one of many possible choices of ω_*ni*_. With various choices for ω_*ni*_, Equation 2 can describe many psychometric models, such as the 4-, 3-, 2-, and 1-parameter logistic models (Birnbaum, 1968; Barton and Lord, 1981), the (non-compensatory) multicomponent latent trait (MLT) model (Embretson, 1984), the deterministic-input, noisy “or” gate model (Templin and Henson, 2006), and the DINA model.

In CDMs, deterministic logic means that attribute mastery status can be known with certainty (i.e., either mastery or non-mastery), and the attributes are applied without stochasticity to produce correct or incorrect latent responses (Rupp et al., 2010), which means that *x*_*nk*_ = α_*nk*_ ∈{0, 1} and ω_*ni*_ = η_*ni*_ ∈{0, 1}. Incorporating the conjunctive condensation rule into Equation (2) creates the deterministic-input, noisy conjunctive model, which is commonly known as the DINA model, as follows:

$$\begin{array}{r} p_{ni1}=g_{i}+\left(1-s_{i}-g_{i}\right)\prod_{k\;=\;1}^{K}\alpha_{nk}^{q_{ik}}. \end{array}$$

According to the deterministic nature of η_*ni*_, *s*_*i*_ and *g*_*i*_ can be defined as *s*_*i*_ = *P*(*Y*_*ni*_ = 0|η_*ni*_ = 1) and *g*_*i*_ = *P*(*Y*_*ni*_ = 1|η_*ni*_ = 0). Moreover, to account for the associations among the attributes and also to reduce the number of latent structural parameters, a higher-order latent structural model can be imposed to create the higher-order deterministic input, noisy “and” gate (HO-DINA) model (de la Torre and Douglas, 2004). The DINA and HO-DINA models classify examinees into two categories. If there is a high degree of uncertainty in the binary classification, the examinees are forced to be classified as either masters or non-masters, usually depending on whether the posterior probability of mastery (given the data) is greater than 0.5 (Karelitz, 2008). The attribute profile report for each examinee from the DINA or HO-DINA model is a vector of zeros or ones specifying the binary status of each attribute.

## The PINC model and its higher-order extension

### The PINC model for independent attributes

In the simplest version, attributes are assumed to be independent of one another (Chen et al., 2012; Li et al., 2015). Let δ_*nk*_ be the probability of mastering the attribute *k* for person *n*, which is assumed to follow a beta distribution:

$$\begin{array}{r} \delta_{nk}\sim \text{Beta}\left(a_{\delta },b_{\delta }\right) \end{array}$$

where *a*_δ_ and *b*_δ_ are the scale parameters. The beta density function can take very different shapes depending on the values of *a*_δ_ and *b*_δ_. For example, when *a*_δ_ = *b*_δ_ = 1, it follows a uniform distribution; when *a*_δ_ > 1 and *b*_δ_ > 1, it follows a unimodal distribution; when *a*_δ_ < 1 and *b*_δ_ < 1, it follows a U-shaped distribution; when *a*_δ_ ≥ 1 and *b*_δ_ < 1, it follows a J-shaped distribution with a left tail; when *a*_δ_ < 1 and *b*_δ_ ≥ 1, it follows a J-shaped distribution with a right tail. Let δ_*n*_ = (δ_*n*1_, δ_*n*2_, …, δ_*nK*_)′ be the probabilistic profile across *K* attributes for person *n*, which is used to produce a probabilistic latent response to item *i* for person *n*, denoted as ρ_*ni*_. Using the conjunctive condensation rule, the relationship between ρ_*ni*_ and δ_*nk*_ can be expressed as follows:

$$\begin{array}{r} \rho_{ni}=\prod_{k\;=\;1}^{K}\delta_{nk}^{q_{ik}}, \end{array}$$

If one of the δ_*nk*_ values is small, ρ_*ni*_ will be small, which means that the attributes are conjunctive.

Incorporating Equation (5) into Equation (2) (i.e., ω_*ni*_ = ρ_*ni*_) creates a PINC model as follows:

$$\begin{array}{r} p_{ni1}=g_{i}+\left(1-s_{i}-g_{i}\right)\prod_{k\;=\;1}^{K}\delta_{nk}^{q_{ik}}, \end{array}$$

where *s*_*i*_ = *P*(*Y*_*ni*_ = 0|lim(ρ_*ni*_) = 1) is the probability of an incorrect response to item *i* if all the required attributes have high mastery probabilities; *g*_*i*_ = *P*(*Y*_*ni*_ = 1|lim(ρ_*ni*_) = 0) is the probability of a correct response to item *i* when at least one of the required attributes has a low mastery probability. These two item-level aberrant response parameters jointly define the observed responses.

Assuming local independence, the likelihood of the observed item responses in the PINC model can be expressed as follows:

$$\begin{array}{r} P\left(\text{Y}|\delta ,\Omega \right)=\overset{N}{\underset{n\;=\;1}{\prod }}\overset{I}{\underset{i\;=\;1}{\prod }}p_{ni1}^{Y_{ni}}{\left(1-p_{ni1}\right)}^{1-Y_{ni}}, \end{array}$$

where *p*_*ni*__1_ is defined in Equation (6).

### The HO-PINC model for correlated attributes

Attributes that are measured by a test are often conceptually related and statistically correlated (de la Torre and Douglas, 2004; Rupp et al., 2010), so it would be helpful to formulate a higher-order structure to link the correlated attributes. de la Torre and Douglas (2004) posited a higher-order latent structural model to account for the associations among attributes as follows:

$$\begin{array}{r} P\left(\alpha_{nk}=1|\theta_{n},\Psi_{k}\right)=\frac{\exp\left(\lambda_{k}\theta_{n}-\beta_{k}\right)}{1+\exp\left(\lambda_{k}\theta_{n}-\beta_{k}\right)}, \end{array}$$

Where **Ψ**_*k*_ = (λ_*k*_, β_*k*_)′ is a vector of the attribute slope and intercept parameters for attribute *k*; θ_*n*_ is the higher-order latent trait, and is assumed to follow the standard normal distribution for model identification. It can be seen that the higher the θ value, the higher the probability of mastering attribute *k* (assuming a positive slope). A combination of Equations (3, 8) creates the HO-DINA model (de la Torre and Douglas, 2004).

Although Equation (8) was developed for CDMs with deterministic logic, that is, α_*nk*_ ~ Bernoulli(*P*(α_*nk*_ = 1)), it can be easily adapted to CDMs with probabilistic logic as follows:

$$\begin{array}{r} \delta_{nk}=\frac{\exp\left(\lambda_{k}\theta_{n}-\beta_{k}\right)}{1+\exp\left(\lambda_{k}\theta_{n}-\beta_{k}\right)}, \end{array}$$

Based on the conjunctive condensation rule, the relationship between ρ_*ni*_ and δ_*nk*_ can be expressed as follows:

$$\begin{array}{r} \rho_{ni}=\prod_{k\;=\;1}^{K}\delta_{nk}^{q_{ik}}=\prod_{k\;=\;1}^{K}{\left[\frac{\exp\left(\lambda_{k}\theta_{n}-\beta_{k}\right)}{1+\exp\left(\lambda_{k}\theta_{n}-\beta_{k}\right)}\right]}^{q_{ik}} \end{array}$$

Combining Equations (2) (let ω_*ni*_ = ρ_*ni*_) and (10) creates the HO-PINC model, which can be presented as follows:

$$\begin{array}{r} p_{ni1}=g_{i}+\left(1-s_{i}-g_{i}\right)\prod_{k\;=\;1}^{K}{\left[\frac{\exp\left(\lambda_{k}\theta_{n}-\beta_{k}\right)}{1+\exp\left(\lambda_{k}\theta_{n}-\beta_{k}\right)}\right]}^{q_{ik}} \end{array}$$

Assuming local independence, the likelihood of the observed item responses in the HO-PINC model can be expressed as follows:

$$\begin{array}{r} P\left(\text{Y}|\delta ,\Omega \right)\equiv P\left(\text{Y}|\theta ,\Psi ,\Omega \right)=\overset{N}{\underset{n\;=\;1}{\prod }}\overset{I}{\underset{i\;=\;1}{\prod }}p_{ni1}^{Y_{ni}}{\left(1-p_{ni1}\right)}^{1-Y_{ni}}, \end{array}$$

where *p*_*ni*__1_ is given in Equation (11).

### Comparison with the DINA and the MLT models

The DINA and MLT models have been commonly used for cognitive diagnoses, so a comparison between the new models and these two relevant models may help to illuminate the new models. The PINC and HO-PINC models can be viewed as fully probabilistic models that simultaneously consider both randomnesses at the item level (in terms of the slip and guessing parameters) and the attribute level (in terms of probabilistic classification). The main difference between the PINC and DINA models is that the former model adopts δ_*nk*_ to account for the probability of mastering attribute *k* for person *n*, whereas the latter adopts α_*nk*_ to indicate whether person *n* masters attribute *k* (either 1 or 0). The attributes in the PINC and HO-PINC models are binary in nature, though they follow the probabilistic logic, whereas the latent traits in the MLT model are continuous in nature on the logit scale. There is one higher-order latent trait to link correlated attributes in the HO-PINC model, whereas there are multiple latent traits but no higher-order structure in the MLT model. In the PINC and HO-PINC models, different items have different item-level aberrant parameters (*s*_*i*_ and *g*_*i*_), whereas in the MLT model, all the items share the same aberrant responses (e.g., multiple-choice items with five options have a lower guessing probability than items with four options), which may be too stringent.

## Bayesian parameter estimation via JAGS

Parameters in the new models can be estimated via the Bayesian approach with the Markov chain Monte Carlo (MCMC) method. In this study, the JAGS (Version 4.2.0; Plummer, 2015) and R2jags packages (Version 0.5-7; Su and Yajima, 2015) in R (Version 3.4 64-bit; R Core Team, 2016) were used to estimate the parameters. JAGS uses a default option of the Gibbs sampler (Gelfand and Smith, 1990) and offers a user-friendly tool for constructing Markov chains for parameters, so the derivation of the joint posterior distribution of the model parameters becomes attainable.

For the PINC model, let *P*(**δ**) be the prior distribution of the probability of mastery, *P*(**Ω**) the prior distribution of the item parameters, and *P*(**Y** | **δ**, **Ω**) the likelihood of the response data (see Equation 7). The posterior distribution of the model parameters is proportional to the prior distribution of the model parameters and the likelihood of the item responses and can be expressed as follows:

$$\begin{array}{r} P\left(\delta ,\Omega |\text{Y}\right)\propto P\left(\text{Y}|\delta ,\Omega \right)P\left(\delta \right)P\left(\Omega \right) \end{array}$$

A non-informative prior distribution is used: δ_*nk*_ ~ Beta (1, 1).

For the HO-PINC model, let *P*(**θ**) be the prior distribution of the general latent trait, *P*(**Ψ**) the prior distribution of the attribute slope and intercept parameters, *P*(**Ω**) the prior distribution of the item parameters, and *P*(**Y** | **θ**, **Ψ**, **Ω**) the likelihood of the response data (see Equation 12). The posterior distribution of the model parameters is expressed as follows:

P(δ,Ω|Y)≡P(θ,Ψ,Ω|Y)∝P(Y|θ,Ψ,Ω)P(θ)P(Ψ)P(Ω)

Specifically, we set θ_*n*_ ~ Normal (0, 1), λ_*k*_ ~ Normal (0, 4), *I*(λ_*k*_ > 0), and β_*k*_ ~ Normal (0, 4) in the following simulation studies and real data analysis.

The same prior distributions for the item parameters were used for the PINC and HO-PINC models. Imposing the monotonicity restriction that *g*_*i*_ < 1 – *s*_*i*_ for all items, the non-informative priors of the item parameters (Culpepper, 2015) are specified as follows: *s*_*i*_ ~ Beta(1, 1) and *g*_*i*_ ~ Beta(1, 1) *I*(*g*_*i*_ < 1 – *s*_*i*_). The corresponding JAGS code for the PINC and HO-PINC models is provided in the Appendix.

## Simulation studies

### Design and date generation

Simulation studies were conducted to evaluate the parameter recovery of the PINC and HO-PINC models, in which the data were simulated from the PINC and HO-PINC models and analyzed with the corresponding data-generating model. There were five attributes. In the PINC model, δ_*nk*_ was generated from a uniform distribution: δ_*nk*_ ~ Beta (1, 1). In the HO-PINC model, θ ~ *N*(0, 1), λ_*k*_ = 1.5 for all attributes, β_1_ = −1, β_2_ = −0.5, β_3_ = 0, β_4_ = 0.5, and β_5_ = 1. Then, each δ_*nk*_ can be calculated according to Equation (9).

**Figure 1:**
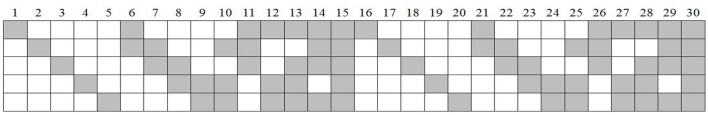
Q'-matrix for 30 items and 5 attributes in the simulation study. Blank means 0 and gray means 1; the first 15 items are used when *I* = 15.

With reference to previous studies (e.g., de la Torre and Douglas, 2004; de la Torre, 2009; de la Torre et al., 2010; Culpepper, 2015; Zhan et al., 2016, 2018a), three independent variables were manipulated, including (a) sample size (*N*): 500 and 1000 examinees; (b) test length (*I*): 15 and 30 items; and (c) item quality (*IQ*): high (*s*_*i*_ = *g*_*i*_ = 0.1) and low (*s*_*i*_ = *g*_*i*_ = 0.2) levels. For high *IQ*, 1 – *s*_*i*_ – *g*_*i*_ = 0.8, which means that the items provide more diagnostic information; for low *IQ*, 1 – *s*_*i*_ – *g*_*i*_ = 0.6, which means that the items provide less diagnostic information. Setting the *s*- and *g*-parameters equally across the items made their impact clear. The Q-matrix is given in Figure 1. The Q-matrix indicates that items 1 to 5 and 16 to 20 measured one attribute; items 6 to 10 and 21 to 25 measured two attributes; item 11 to 15 and 26 to 30 measured three or more attributes. Thirty replications were implemented in each condition.

### Analysis

In each replication, two Markov chains (*n.chain* = 2) with random starting points were used, and each chain ran 10,000 iterations (*n.iter* = 10,000), with the first 5,000 iterations in each chain as burn-in (*n.burn* = 5,000). Without thinning interval (*n.thin* = 1). Finally, the remaining *n.chain* ^*^ (*n.iter* – *n.burn*) / *n.thin* = 10,000 iterations for the model parameter inferences. The potential scale reduction factor (PSRF; Brooks and Gelman, 1998) was computed to assess the convergence of each parameter. The values of the PSRF less than 1.1 or 1.2 indicate convergence (Brooks and Gelman, 1998; de la Torre and Douglas, 2004). Our studies indicated that the PSRF was generally less than 1.01, suggesting good convergence. Specifically, when we encounter a non-convergent dataset (with the rule of PSRF < 1.2), we will replace this dataset with a new one. This procedure will continue until all monitored parameters in all datasets under all conditions achieve the convergence. The root mean square error (RMSE) and the correlation between the generated values and estimated values (Cor) for the parameters were computed to evaluate the parameter recovery.

## Results

### Recovery of model parameters in the PINC model

**Figure 2:**
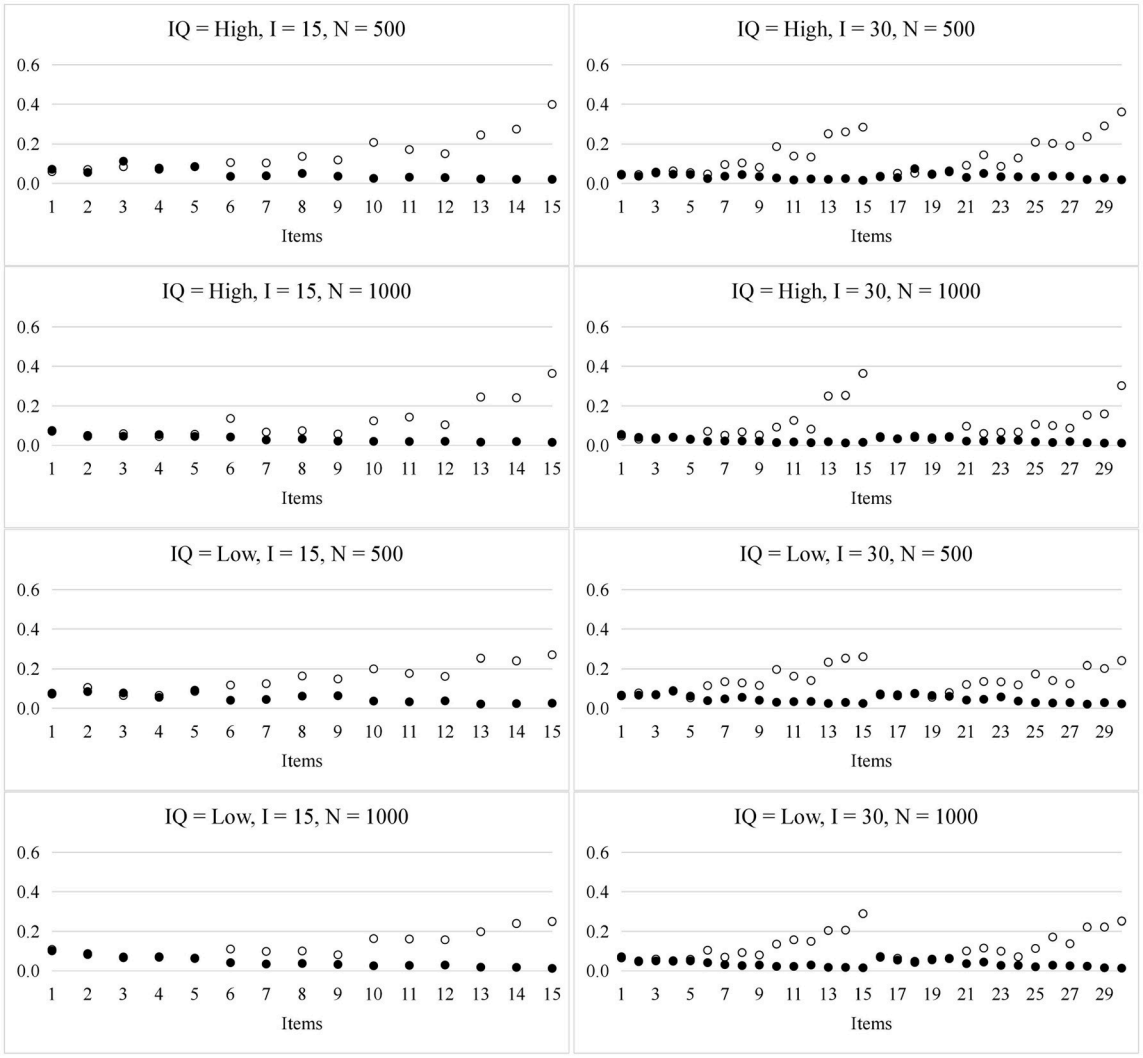
RMSE for the item parameters in the PINC model. ◦ represents *s*_*i*_ and • represents *g*_*i*_; *IQ*, item quality; *N*, sample size; *I*, test length.

The plot in Figure 2 shows the RMSE for the item parameters. As in prior studies (de la Torre, 2009; Culpepper, 2015), the sampling variability for the *s*_*i*_ and *g*_*i*_ parameters was associated with the number of required attributes. For example, according to the Q-matrix, there is one required attribute in the first five items and two required attributes in the next four items (i.e., items 6 to 9), respectively. For the *s*_*i*_ parameters, the RMSEs of the first five items were smaller than those of the next four items; In contrast, for the *g*_*i*_ parameters, the RMSEs of the first five items were a little bit larger than those of the next four items. Overall, the larger the number of attributes required by an item, the larger the RMSE for *s*_*i*_, but the smaller the RMSE for *g*_*i*_. Such results were expected because the number of persons who mastered all the required attributes with a high probability decreased as the number of required attributes increased, so the variability of *s*_*i*_ increased. In contrast, the number of persons who mastered any of the required attributes with a low probability increased as the number of the required attributes increased, so the variability of *g*_*i*_ decreased. Furthermore, the larger the sample size, the smaller the RMSE. The item quality and test lengths had trivial effects on the recovery of the item parameters.

The recovery of the probability of mastery is summarized in Table 1. In general, all the RMSE values were around 0.22, and almost all the Cor values were higher than 0.9 across all the conditions. The longer test length is, the larger sample size is, and the higher item quality would lead to smaller RMSE and larger Cor.

**Table 1:** Recovery of the attribute parameters in the PINC model.

***IQ***	***N***	***I***	**Index**	**δ_1_**	**δ_2_**	**δ_3_**	**δ_4_**	**δ_5_**
High	500	15	RMSE	0.232	0.230	0.224	0.228	0.227
			Cor	0.923	0.938	0.916	0.925	0.920
		30	RMSE	0.210	0.210	0.203	0.201	0.207
			Cor	0.962	0.966	0.950	0.955	0.953
	1,000	15	RMSE	0.229	0.228	0.225	0.229	0.231
			Cor	0.928	0.938	0.932	0.933	0.928
		30	RMSE	0.210	0.204	0.202	0.205	0.209
			Cor	0.956	0.961	0.962	0.958	0.957
Low	500	15	RMSE	0.245	0.245	0.237	0.240	0.240
			Cor	0.898	0.890	0.873	0.909	0.882
		30	RMSE	0.235	0.233	0.226	0.229	0.230
			Cor	0.923	0.941	0.932	0.934	0.932
	1,000	15	RMSE	0.241	0.243	0.239	0.245	0.244
			Cor	0.888	0.896	0.897	0.901	0.893
		30	RMSE	0.231	0.230	0.226	0.231	0.234
			Cor	0.934	0.943	0.936	0.938	0.931

*IQ, item quality; N, sample size; I, test length*.

### Recovery of model parameters in the HO-PINC model

**Figure 3:**
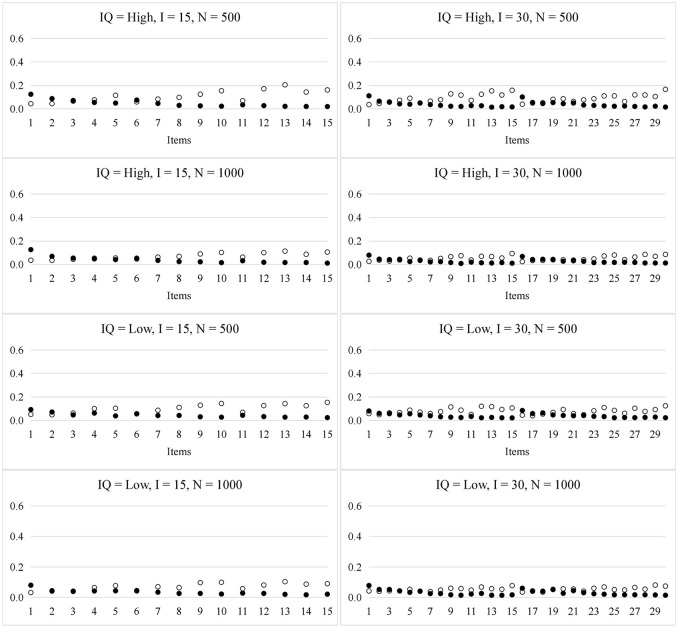
RMSE for the item parameters in the HO-PINC model. ◦ represents *s*_*i*_ and • represents *g*_*i*_; *IQ*, item quality; *N*, sample size; *I*, test length.

Figure 3 presents the RMSE for the item parameters in the HO-PINC model. In general, the recovery of the item parameters was satisfactory and better than that in the PINC model. For example, the sampling variability of *s*_*i*_ for the HO-PINC model was nearly half that for the PINC model, when the items required more than two attributes.

Table 2 summarizes the recovery of the probability of mastery in the HO-PINC model. Overall, the recovery patterns of the person parameters in the HO-PINC model were similar to those for the PINC model. Compared with the PINC model, the RMSE and Cor for the HO-PINC model were closer to 0 and 1, respectively.

**Table 2:** Recovery of the attribute parameters in the HO-PINC model.

***IQ***	***N***	***I***	**Index**	**δ_1_**	**δ_2_**	**δ_3_**	**δ_4_**	**δ_5_**
High	500	15	RMSE	0.158	0.152	0.144	0.141	0.125
			Cor	0.956	0.972	0.984	0.990	0.993
		30	RMSE	0.121	0.112	0.108	0.100	0.094
			Cor	0.976	0.988	0.992	0.995	0.996
	1,000	15	RMSE	0.152	0.142	0.140	0.134	0.123
			Cor	0.952	0.973	0.983	0.991	0.994
		30	RMSE	0.108	0.106	0.104	0.098	0.090
			Cor	0.976	0.987	0.992	0.995	0.997
Low	500	15	RMSE	0.198	0.188	0.190	0.198	0.179
			Cor	0.929	0.956	0.975	0.987	0.990
		30	RMSE	0.153	0.153	0.150	0.140	0.132
			Cor	0.960	0.972	0.981	0.990	0.994
	1,000	15	RMSE	0.184	0.177	0.180	0.172	0.169
			Cor	0.925	0.957	0.973	0.985	0.990
		30	RMSE	0.145	0.142	0.142	0.136	0.126
			Cor	0.954	0.970	0.985	0.989	0.994

*IQ, item quality; N, sample size; L, test length*.

Table 3 presents the RMSE and Cor for the higher-order latent trait in the HO-PINC model. The RMSE ranged from 0.391 to 0.618 across conditions, which was acceptable because the latent trait was measured by only five binary attributes. The results were similar to those found in the literature of the HO-DINA model (de la Torre and Douglas, 2004; Huang and Wang, 2014; Zhan et al., 2018a,b). The longer the test length and the higher the item quality, the smaller the RMSE and the larger the Cor, indicating a better recovery. In addition, in previous studies about the HO-DINA model (e.g., de la Torre and Douglas, 2004; Zhan et al., 2018a,b), the correlation coefficient of the true and estimated higher-order ability is approximately ranged from 0.6 to 0.8; However, in the HO-PINC model, the correlation coefficient is generally higher than 0.95, indicating that the higher-order ability can be better recovered in the HO-PINC model than in the HO-DINA model.

**Table 3:** Recovery of the higher-order latent trait in the HO-PINC model.

***IQ***	***N***	***I***	**RMSE**	**Cor**
High	500	15	0.494	0.968
		30	0.391	0.979
	1,000	15	0.506	0.967
		30	0.400	0.980
Low	500	15	0.618	0.959
		30	0.495	0.969
	1,000	15	0.617	0.957
		30	0.506	0.968

*IQ, item quality; N, sample size; I, test length*.

Overall, the parameter recovery of both the PINC and HO-PINC models was satisfactory. The recovery was better in the HO-PINC model than in the PINC model, which might be because the incorporation of a higher-order structure allowed the information about one attribute to be used in estimating the other attributes. This phenomenon is analogous to the joint estimation of multiple unidimensional tests in which the correlation among latent traits is taken into consideration to improve the parameter estimation of individual dimensions (Wang et al., 2004).

## An empirical example

### Material and data description

A real dataset from the Examination for the Certificate of Proficiency in English (ECPE) was analyzed to demonstrate the applications of the new models. The ECPE measures the advanced English skills of examinees whose primary language is not English (Templin and Hoffman, 2013). A total of 2,922 examinees answered 28 multiple-choice items with three required attributes: α_1_, or morphosyntactic rules; α_2_, or cohesive rules; and α_3_, or lexical rules. The Q-matrix can be found in Templin and Hoffman (2013). According to the description of each attribute, these three attributes appeared to be conceptually related to a general English proficiency, which might justify the use of a higher-order structure.

### Analysis

Four models were fitted and compared: the PINC, HO-PINC, DINA, and HO-DINA models. The number of chains, burn-in iterations, and post-burn-in iterations was consistent with those in the simulation study. Convergence was well achieved (see Figure A1 in Appendix). The deviance information criterion (DIC; Spiegelhalter et al., 2002) and the log conditional predictive ordinate (LCPO; Kim and Bolt, 2007) multiplied by −2 (−2LCPO) were computed for model selection. In the DIC, the effective number of parameters was computed as var(*D*)/2 (Gelman et al., 2003), where $\bar{D}$ is the posterior mean of deviance in the MCMC samples and measures how well the data fit the model using the likelihood function (−2 log-likelihood, −2LL). A smaller value of the DIC and −2LCPO indicates a better fit.

### Results

Among the four models, the HO-PINC model was identified as the best-fitting model based on the DIC and the test-level −2LCPO, as shown in Table 4. The item-level −2LCPO can be used to further examine whether the finding is consistent across items. The HO-PINC model had the smallest item-level −2LCPO value for each item (not presented). In general, the higher-order models (i.e., the HO-DINA and HO-PINC models) had a better fit than their corresponding non-structured counterparts (i.e., the DINA and PINC models), which means that the incorporation of a general English proficiency was justified.

**Table 4:** −2LL, DIC and −2LCPO indices for the ECPE data.

**Model**	**−2LL**	**DIC**	**−2LCPO (test-level)**
PINC	**80233.58**	89829.31	84539.30
HO-PINC	80880.11	**84043.92**	**83233.54**
DINA	81246.47	87608.53	84191.80
HO-DINA	81143.27	86752.73	84167.54

*−2LL, −2 log likelihood; DIC, deviance information criterion; −2LCPO, −2 log conditional predictive ordinate. Bold values are indicated as significance mark*.

**Figure 4:**
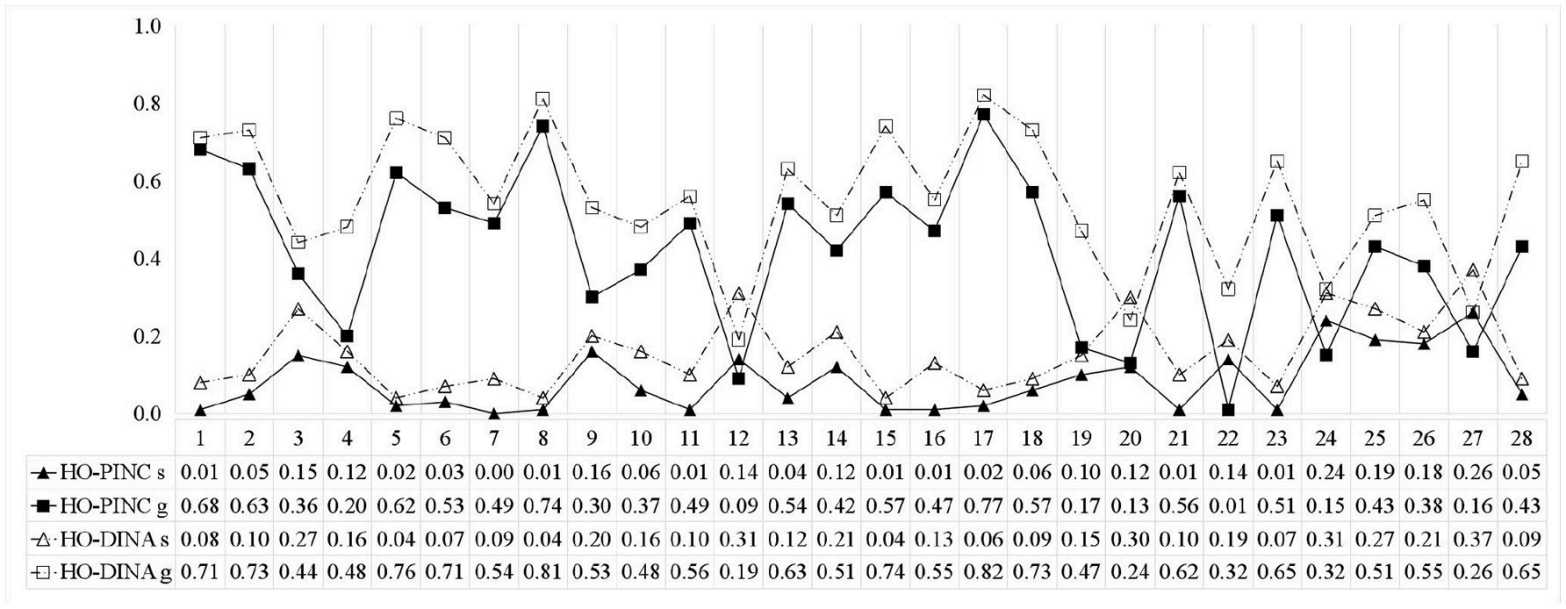
Item parameter estimates for the ECPE data.

Figure 4 shows the item parameter estimates obtained from the HO-PINC and HO-DINA models. Similar to previous studies (e.g., Templin and Hoffman, 2013), many of the estimated *g*_*i*_ values were large, which means that the examinees might utilize some other attributes or skills that were not included in the Q-matrix. In general, the item parameter estimates from the HO-PINC model were smaller than those from the HO-DINA model. A possible explanation is that standard CDMs dichotomize the attribute mastery probabilities to mastery or non-mastery, so examinees with an intermediate status would be forced to be classified, and the uncertainty associated with this classification might contribute to the inflation of the item parameter estimates in the HO-DINA model. In other words, the inherent uncertainty at the attribute level was absorbed into the item level when binary classification was adopted.

Table 5 shows the attribute parameter estimates for four examinees. The variable ${\hat{\delta }}_{nk}$ can distinguish examinees in a finer manner than ${\hat{\alpha }}_{nk}$. A probability of mastery between 0.4 and 0.6 (Hartz, 2002) is shown in bold. For Person 1, the estimated probabilities of the three attributes in the HO-PINC model were all very close to 1, so the binary classifications in the HO-DINA model seemed appropriate. For Person 14, the estimated probabilities in the HO-PINC model were 0.255, 0.455, and 0.593, respectively, and the binary classifications in the HO-DINA model were 0, 0, and 1, respectively. There was a great amount of uncertainty in attributes 2 and 3, but it was ignored by the HO-DINA model.

**Table 5:** Attribute estimates for the ECPE data under the HO-PINC and HO-DINA models.

**Person**	**HO-PINC**	**HO-DINA**
	$\left({\hat{\delta }}_{\;n1},{\hat{\delta }}_{\;n2},{\hat{\delta }}_{\;n3}\right)$	$\left({\hat{\alpha }}_{n1},{\hat{\alpha }}_{n2},{\hat{\alpha }}_{n3}\right)$
1	(0.909, 0.916, 0.978)	(1, 1, 1)
889	(**0.429**, 0.605, 0.768)	(0, 1, 1)
14	(0.255, **0.455**, **0.593**)	(0, 0, 1)
1071	(0.084, 0.246, 0.301)	(0, 0, 0)

*Bold values are indicated as significance mark*.

**Figure 5:**
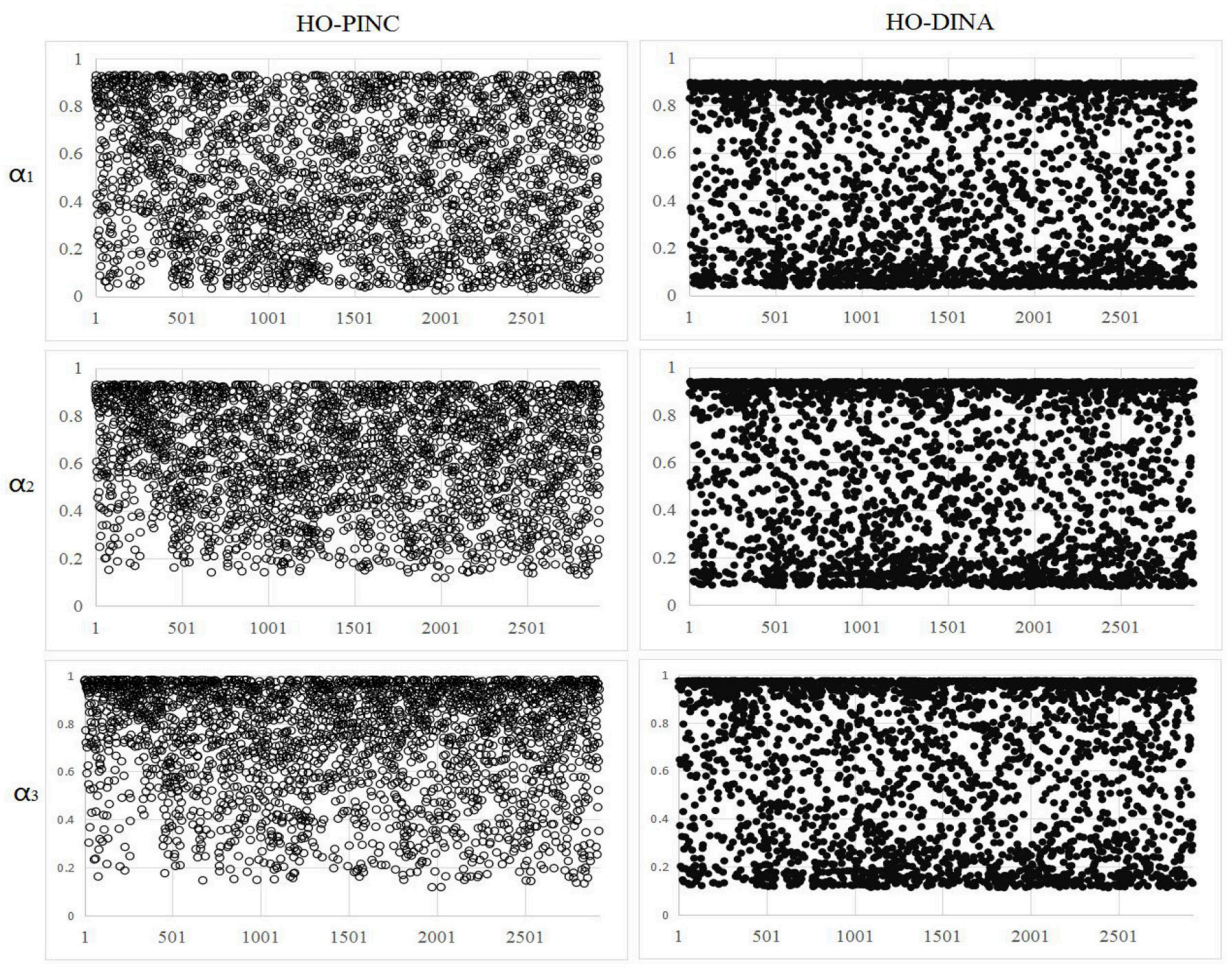
Attribute mastery probability estimates under the HO-PINC and HO-DINA models. ◦ represents the mastery probability of attributes, ${\hat{\delta }}_{nk}$ in the HO-PINC model, and • represents the mastery probability of attributes, $P\left({\hat{\alpha }}_{nk}=1\right)$ in the HO-DINA model.

It should be noted that the HO-DINA model can also provide a mastery probability for each attribute, denoted as $P\left({\hat{\alpha }}_{nk}=1\right)$. Essentially, ${\hat{\delta }}_{nk}$ in the HO-PINC model and $P\left({\hat{\alpha }}_{nk}=1\right)$ in the HO-DINA model are almost the same in mathematical expressions, see Equations (8, 9). The main difference between them is that the $P\left({\hat{\alpha }}_{nk}=1\right)$ needed a Bernoulli transition (i.e., ${\hat{\alpha }}_{nk}\sim \text{Bernoulli}\left(P\left({\hat{\alpha }}_{nk}=1\right)\right)$) before imposing into the item response function, while the ${\hat{\delta }}_{nk}$ can be directly inputted into the item response function. Even though the correlations between ${\hat{\delta }}_{nk}$ and $P\left({\hat{\alpha }}_{nk}=1\right)$ for the three attributes were very high (0.97, 0.96, and 0.94, respectively), there were subtle differences between them, as shown in Figure 5 for the 2,922 examinees. In general, $P\left({\hat{\alpha }}_{nk}=1\right)$ clustered more around the two extremes of 0 and 1, whereas ${\hat{\delta }}_{nk}$ was more uniformly distributed. The variances of ${\hat{\delta }}_{nk}$ for the three attributes were 0.077, 0.048, and 0.051, whereas the variances of $P\left({\hat{\alpha }}_{nk}=1\right)$ for the three attributes were 0.112, 0.108 and 0.106. That is, the variance of ${\hat{\delta }}_{nk}$ was approximately half that of$P\left({\hat{\alpha }}_{nk}=1\right)$.

## Conclusion and discussion

In contrast to deterministic logic, in which a statement such as “a person masters an attribute” can be verified without uncertainty, probabilistic logic acknowledges uncertainty in such a statement using a probabilistic expression. This study developed the PINC model, in which the probability of mastering an attribute for a person is treated as a parameter, and the HO-PINC model, in which a latent trait is further added to account for the associations among the attributes. The results of the simulation study indicated that (a) the parameters for two proposed models can be well recovered by using the proposed Bayesian MCMC method, and (b) imposing a higher-order latent structure among probabilistic attributes can further improve the model parameter recovery. Furthermore, an empirical example was provided to demonstrate the applications of the proposed models. And the results of the empirical example supported the utility of the HO-PINC model, mainly because, in reality, attributes that are measured by a test are often conceptually related and statistically correlated. Overall, according to the results of the simulation study and the empirical example, we recommend using the HO-PINC model in the future. In practice, it is still useful to fit both the new models and the standard CDMs and compare their fit. Probabilistic logic is empirically supported if it has a better fit, and many examinees have a probability of mastery around 0.5.

The work presented in this article is an attempt to apply probabilistic logic to CDMs. Despite promising results, further exploration is needed. First, only the conjunctive condensation rule was employed in this study. Future studies can develop other probabilistic-input models based on other condensation rules (e.g., disjunctive or compensatory), or create a general framework to include general probabilistic-input CDMs, such as those performed by von Davier (2008), Henson et al. (2009), and de la Torre (2011). Second, the new models focused on dichotomous items. It is important and practical to adapt the models to polytomous items (von Davier, 2008; Ma and de la Torre, 2016) and mixed-format tests. Third, throughout this study, it was assumed that there were only two categories (mastery or non-mastery) in each attribute. It would be interesting in future work to develop CDMs for polytomous attributes Karelitz (2004) with probabilistic logic. Fourth, it is possible that some attributes are prerequisites to the mastery of other attributes; that is, attributes can have a hierarchical structure (Leighton et al., 2004). Future studies should take attribute hierarchies into account in the proposed models. Finally, recent developments in the assessment of differential item functioning (Li and Wang, 2015) or local item dependence (Zhan et al., 2015) in CDMs could be conducted on the PINC or HO-PINC models.

## Author contributions

PZ contributed to the conception, design, and analysis of data as well as paper drafting and revising the manuscript. W-CW contributed to conception, design, and revising the manuscript. HJ contributed to the design and critically revising the manuscript. YB contributed to the critically revising the manuscript.

### Conflict of interest statement

The authors declare that the research was conducted in the absence of any commercial or financial relationships that could be construed as a potential conflict of interest.
