# Supplementary material for: Probabilistic-Input, Noisy Conjunctive Models for Cognitive Diagnosis
Source: Front Psychol. 2018 Jun 14;9:997. doi: 10.3389/fpsyg.2018.00997 (PMC6010692; doi:10.3389/fpsyg.2018.00997)
Supplement: Supplementary file 1 [file Presentation_1.pdf]

Appendix: JAGS code for the PINC and HO-PINC models

```
####################
###The PINC model###
####################
PINC.model
{
  for (n in 1:N) {
    for (k in 1:K) {delta[n, k] ~ dbeta(1, 1)}
                    }
  for (n in 1:N) {
    for (i in 1:I) {
       for (k in 1:K) {w[n, i, k] <- pow(delta[n, k], Q[i, k])}
       rho[n, i] <- prod(w[n, i, ])
       p[n, i] <- g[i] + (1 - s[i] - g[i]) * rho[n, i]
       Score[n, i] ~ dbern(p[n, i])
                      }}
  for (i in 1:I) {
    s[i] ~ dbeta(1, 1)
    g[i] ~ dbeta(1, 1)  T(0, 1-s[i])}}


#######################
###The HO-PINC model###
#######################
HO-PINC.model
{
  for (n in 1:N) {
    for (k in 1:K) {logit(delta[n, k]) <- lamda[k] * theta[n] - beta[k]}
    theta[n] ~ dnorm(0, 1)
    }
  for (k in 1:K) {
    beta[k] ~ dnorm(0, 0.25)
    lamda[k] ~ dnorm(0, 0.25)  T(0, )
    }
  for (n in 1:N) {
    for (i in 1:I) {
      for (k in 1:K) {w[n, i, k] <- pow(delta[n, k], Q[i, k])}
      rho[n, i] <- prod(w[n, i, ])
      p[n, i] <- g[i] + (1 - s[i] - g[i]) * rho[n, i]
      Score[n, i] ~ dbern(p[n, i])
                      }}
    for (i in 1:I) {
        s[i] ~ dbeta(1, 1)
        g[i] ~ dbeta(1, 1)  T(0, 1-s[i])}}
```

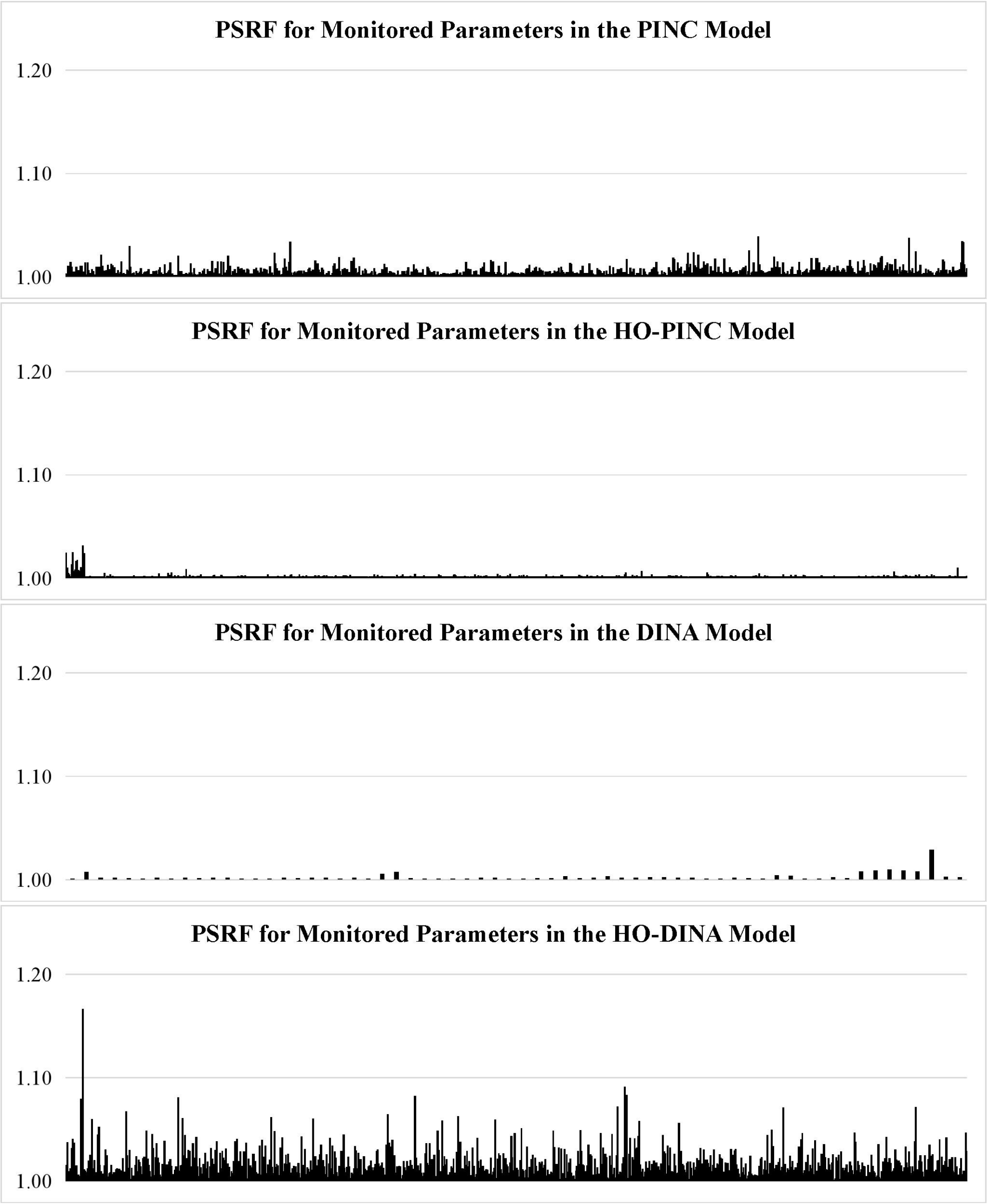

Figure A1. Potential scale reduction factor for monitored parameters in real data study.

Note: for the PINC model, PSRF assesses convergence of item parameters and probabilistic attributes; for HO-PINC model, PSRF assesses convergence of item parameters, higher-order latent structural parameters, and higher-order abilities. As deterministic attributes in the DINA and HO-DINA models are categorical variables, PSRF is not suitable for them. Thus, for the DINA model, PSRF assesses convergence of item parameters and mixture proportions; for the HO-DINA model, PSRF assesses convergence of item parameters, higher-order latent structural parameters, and higher-order abilities.
